# Interaction Between Social Support and Muscle Dysmorphia: The Role of Self-Efficacy and Social Media Use

**DOI:** 10.3390/bs15020122

**Published:** 2025-01-24

**Authors:** Fiorenza Giordano, Danilo Calaresi, Loriana Castellani, Valeria Verrastro, Tommaso Feraco, Valeria Saladino

**Affiliations:** 1 Department of Human Sciences, Society and Health, University of Cassino and Southern Lazio, 03043 Cassino, Italy; loriana.castellani@unicas.it; 2 Department of Health Sciences, University “Magna Graecia” of Catanzaro, 88100 Catanzaro, Italy; danilo.calaresi@unicz.it (D.C.); valeria.verrastro@unicaz.it (V.V.); valeria.saladino@unicz.it (V.S.); 3 Department of General Psychology, University of Padova, 35131 Padua, Italy; tommaso.feraco@unipd.it

**Keywords:** muscle dysmorphia, social support, self-efficacy, social media use, adolescents, young adults

## Abstract

Muscle dysmorphia (MD) consists of a type of body dysmorphic disorder and involves a distorted perception of one’s muscles, strict diets, and workouts. Mostly, studies focus on adult male athletes, especially bodybuilders, while research on adolescents and women is limited. Our study aims to explore potential protective or risk factors influencing MD, using a mediation model calculated through Structural Equation Modeling (SEM) and at the same time help to involve female individuals in the exploration of a distress traditionally and predominantly analyzed only in male individuals. The model examines whether problematic social media use (PSMU) and generalized self-efficacy (GSE) are potential first- and second-level mediators, respectively, in the relationship between perceived social support (PSS) and MD. The sample consisted of 2325 individuals of both sexes aged 14–29 years. Structural equation models were used to assess effect sizes, regressions, and direct and indirect effects of perceived social support on muscle dysmorphia and general self-efficacy both on problematic social media use and muscle dysmorphia. Our results suggest that inadequate perceived social support may reduce individuals’ perceived effectiveness in managing daily challenges, potentially leading to problematic use of social media, which may contribute to muscle dysmorphia symptoms. Future interventions could promote a healthier perception of one’s body, improving confidence in individuals’ coping strategies and strengthening the social environment of reference.

## 1. Introduction

Muscle Dysmorphia has been officially classified as a subtype of body dysmorphic disorder in the *Diagnostic and Statistical Manual of Mental Disorders 5th ed.* constituted by a distorted perception of one’s body (American Psychiatric Association, 2013; Cooper et al., 2020; Underwood & Olivardia, 2023). Individuals who suffer from MD do not perceive themselves as sufficiently toned and muscular (Hellfeldt et al., 2019; Santarnecchi & Dèttore, 2012). They are obsessed with their physical appearance and musculature and resort to any strategy—sometimes even taking anabolic–androgenic steroids—to achieve an ideal aesthetic model (Imperatori et al., 2022; Tovt & Kajanová, 2021). Furthermore, muscle dysmorphia is characterized by the implementation of strict diets and rigorous and strenuous daily workouts (Di Fabio & Busoni, 2008). These conducts are carried out even in the presence of fatigue, injury, and discomfort and might cause physical damage and social or work impairment (Tovt & Kajanová, 2021). In this framework, problematic social media use (PSMU) (Monacis et al., 2017; Sommantico et al., 2023) emerges in the literature as one of the most common dysfunctional behaviors related to body image dissatisfaction and the wide range of related disorders, such as negative evaluation of one’s physical appearance, eating disorders, obsessive–compulsive disorder in the form of body dysmorphia, and muscle dysmorphia (Griffiths et al., 2018; Imperatori et al., 2022; Rizwan et al., 2022; Ryding & Kuss, 2020). Regarding the definition of problematic use of social media, since it is not currently mentioned in the *Diagnostic and Statistical Manual of Mental Disorders* (5th edition, text revision; DSM-5-TR) (American Psychiatric Association, 2013) no uniformly accepted definition exists, and consequently, there is a wide and heterogeneous range of terminology referring to it as “addiction”, “excessive use”, ”misuse”, “pathological use”, and “problematic use”. Despite the lack of agreement in recognizing, describing, and defining this distress, most scholars agree that problematic use of social media is a potential behavioral addiction specifically related to the misuse or overuse of new technologies, such as Internet addiction, smartphone addiction, and online gaming addiction, recognizing as its constituent characteristics the six criteria identified by Griffiths (2008) in his study on behavioral addictions: mood modification (i.e., change in emotional states due to engagement in social media use); salience (i.e., intense preoccupation with social media use); tolerance (i.e., increasingly frequent social media use); withdrawal symptoms (i.e., emotional and physical symptoms due to limited or interrupted social media use); conflict (i.e., intrapsychic and interpersonal problems due to social media use); and relapse (i.e., rapid return to excessive social media use after a period of abstinence) (Ryding & Kuss, 2020; Sommantico et al., 2023). Another factor that emerges in many studies is the relationship between problematic social media use, lower life satisfaction, and poor mental health in general, in the form of maladaptive cognitions, such as self-criticism and dysfunctional attitudes, decreased self-esteem and negative self-concept, and increased risk of rumination, as well as in the form of risk or maintenance factors for a multitude of psychological stresses, including depression, anxiety, loneliness, and social isolation, in addition to those already mentioned related to body image perception and eating disorders (Imperatori et al., 2022; Meshi & Ellithorpe, 2021; Rizwan et al., 2022; Ryding & Kuss, 2020; Sommantico et al., 2023). In accordance with the aforementioned assumptions, in our study on the problematic use of social media, we mean behavior that is beyond the control of individuals, that creates anxiety and distress when it cannot be acted upon, or difficulty in stopping it, or a tendency to prolong it much longer than expected, with a general impairment of social relationships, school and work performance, and a reduction or lack of other interests outside of social media platforms. Based on these considerations, we decided not to focus our research on specific social media platforms or set a specific time interval for their use. Social media use allows people, especially younger people, to connect with each other and communicate, receiving social rewards by representing one of the most important instruments of communication, socialization, and comparison (Imperatori et al., 2022; Li, 2019). As a result, social media’s overemphasis on an individual’s outward appearance could increase the sense of body image dissatisfaction, i.e., negative appraisal of one’s physical appearance. Indeed, several online platforms, of which TikTok, Facebook, Instagram, etc., promote an idealized body image and rigid beauty and physical fitness, are becoming a source of discomfort and distress, especially for adolescents and young adults. Studies on emerging trends among youths suggest that posts on Social Media (SM) (e.g., Facebook, Instagram) identified with the hashtag “thinspiration” contribute to idealizing extremely thin bodies. Exposure to these contents is associated with beliefs, attitudes, and symptoms often related to eating disorders (Griffiths et al., 2018; Imperatori et al., 2022; Rizwan et al., 2022). Similarly, it has been hypothesized that the increasing number of posts identified with the hashtag “fitspiration” (i.e., content shared to promote healthy messages focused on fitness, exercise, eating styles, physical appearance, and weight control) may emphasize an unrealistic body image and unattainable habits, thus leading to negative feelings in relation to mood, body image, and emotional well-being (Griffiths et al., 2018; Mancin et al., 2024; Schoenenberg & Martin, 2020). The literature suggests that although gender differences may exist in relation to the idealized body type proposed by social media, it should be emphasized that dysfunctional use of such models may be associated with the development and maintenance of body image dissatisfaction in both genders equally. In addition, other studies hypothesize that the use of social networks in comparing physical appearance may promote the development and maintenance of symptoms of body dysmorphic disorder and should be considered an important risk factor (Imperatori et al., 2022; Mancin et al., 2024; Rizwan et al., 2022).

The literature identifies two key factors that serve as protective measures against heterogeneous psychological distress: perceived social support and general self-efficacy. These findings align with the tenets of social cognitive theory and social comparison. The tenets of social cognitive theory posit that the observation of exemplary conduct can positively influence observers’ task and job performance. This is because observational learning from role models can enhance self-efficacy. Social comparison theory posits that the perception of higher performance is accompanied by negative self-evaluations, which, in many circumstances, should reduce self-efficacy and subsequent task and job performance (Gerber et al., 2018). In particular, social comparison theory is concerned with the psychological processes by which individuals evaluate themselves in comparison to others. The topic of social comparison has been a prominent area of research in the fields of social and personality psychology since 1954. The theory has subsequently undergone significant expansion, with links now established to social cognition, individual differences, developmental psychology, and experimental social psychology. The primary objective is to examine the processes through which individuals engage in social comparison, the motivations underlying these comparisons, the identity of those who engage in such comparisons, and the consequences of these comparisons (Haddad, 2022).

On the basis of this theory, we decided to include perceived social support and general self-efficacy in our research in order to investigate their potential role as predictor and mediator, respectively in the development of problematic social media use and muscle dysmorphia discomfort. Self-efficacy is situated within the broader theoretical framework of social cognitive theory (Bandura et al., 2003) and consists of personal perceptions of problem-solving skills and coping strategies for managing stressful or challenging events (Waddington, 2023). Perceived self-efficacy represents a positive sense of personal competence (Schwarzer & Jerusalem, 1995) and plays a key role in determining behavior, goals, and overall human functioning. Based on Bandura’s social cognitive theory, self-efficacy is a personal construct that influences and is influenced by behaviors and social/environmental variables. According to this theory, human functioning depends on three main interacting factors: personal, social/environmental, and behavioral, each of which influences and is influenced by the others (Schwarzer & Jerusalem, 1995). According to Bandura et al. (2003) there are two main types of expectations: self-efficacy expectations, which refer to one’s personal ability to achieve a desired outcome, and outcome expectations, which refer to the likelihood of achieving a desired outcome (Bandura et al., 2003; Tortosa Martínez et al., 2024). Bandura defines the former as judgments about the ability to perform certain actions to achieve a goal or to deal effectively with stressful situations (Bandura et al., 2003). The researcher’s concept of self-efficacy emphasizes that an individual’s belief in his or her own abilities can significantly influence his or her approach to goals, tasks, and challenges. For instance, students who have high self-efficacy believe that they can perform well and achieve their goals (Iwanowicz-Palus et al., 2021; McLean et al., 2023; Meshi & Ellithorpe, 2021; Tortosa Martínez et al., 2024). This positive belief system acts as a protective factor against the negative impacts of social appearance anxiety and body image dissatisfaction (Bentler & Bonett, 1980). Consequently, low self-efficacy appears to be related to a higher incidence of anxiety and depression symptoms and lower levels of subjective well-being (Ganson et al., 2023; Karr & White, 2024; Lippke, 2020). In contrast, high self-efficacy enables individuals to overcome fear and anxiety related to their social appearance, allowing them to focus on their strengths and abilities, improve stress regulation, increase self-esteem, well-being, and physical health, and become more resilient to acute and chronic illnesses (Dinh & Bonner, 2023).

Perceived social support (PSS) is an important factor related to general self-efficacy (Iwanowicz-Palus et al., 2021; McLean et al., 2023). Social support is a multidimensional concept that refers to social integration and interpersonal relationships that impact individuals by enabling them to feel surrounded by people they can rely on. More specifically, it can be defined as the social and psychological support that an individual receives or perceives as available from family, friends, and his or her community and includes emotional and instrumental support. In other words, PSS consists of the perception of being cared for, protected, respected, and valued by others and of being recognized and treated as an integral part of a social network (Iovino et al., 2023). This support can be provided by family members, friends, relatives, colleagues, and significant others. Through this process, individuals observe, imitate, and reinforce certain behaviors to increase their chances of being accepted and appreciated (Sibilia et al., 1995). The processes involved in social support are strongly related to mental and physical health, as they promote well-being and life satisfaction and increase resilience by acting as protective factors from heterogeneous psychological distress, from anxiety to depressive symptoms, and from body image-related distress to addictions (Iovino et al., 2023). There is substantial evidence highlighting the role of social support in promoting psychological health. Research has shown that social support influences health directly and indirectly through cognitive mechanisms, coping strategies, and health behaviors, demonstrating that individuals with supportive families or friends enjoy better health and recover faster from health problems and traumatic events than those who are less socially integrated. In fact, it has long been shown that social support is a moderator of stress, functions as an effective coping mechanism for dealing with stressors, and contributes significantly to health maintenance, disease prevention, and medical treatment success (Meshi & Ellithorpe, 2021; Xie & Kim, 2022). The presence of another person in a problematic situation reduces stress and makes one feel safer and in control. In addition, the literature suggests that the perception of one’s social support network has an essentially greater coping function and exerts a stronger effect on mental health than actual social support received. The perception of having social support available may provide a buffer in times of stress, increase happiness, and improve psychological well-being. Regarding the relationship between perceived social support and social media as a communication and relationship-building tool, many scholars point out that social support in real life is associated with a reduction in depressive symptoms and greater overall well-being, while social support provided through social media does not appear to influence well-being and may increase depressive and anxiety symptoms. These researchers found that the greater the problematic use of social media, the lower the perceived social support in real life and the worse the mental health (Hossain et al., 2024; Iovino et al., 2023; Iwanowicz-Palus et al., 2021; Lin et al., 2021; McLean et al., 2023; Meshi & Ellithorpe, 2021; Saladino et al., 2024).

In accordance with the literature, which recognizes perceived social support as playing an important role in enhancing individuals’ physical and psychological well-being, our study aims to examine this variable in the exploration of the phenomenon of muscle dysmorphia in adolescents and young adults of both sexes, testing it as a direct (Calaresi et al., 2024; Lovely Professional University et al., 2016; Monacis et al., 2017) and indirect predictor via GSE and sequentially via PSMU of MD-related symptomatology (Bentler & Bonett, 1980). The hypothesis underlying the selected model consists of the assumption, derived from the literature, that the perception of inadequate social support may have a negative impact on the psychosocial and emotional growth of adolescents and young people, reducing their coping skills and resources and making them use dysfunctional ways of communicating and confronting others. On the basis of the literature reviewed, which recognizes its key role in determining and influencing human behavior, choices, and attitudes in achieving goals and coping with stressful and problematic events, we propose to examine the potential mediation of general self-efficacy in the aforementioned relationship. Finally, we also propose to test the role of problematic use of social media as another mediating factor, as we hypothesize, on the basis of the literature, that it may turn out to be a risk factor in the symptomatology of muscular dysmorphia, because its specific characteristics may make it a substitute for social relationships and communication in the real world, and at the same time, as a communication tool that places great importance on appearance and promotes the image of an increasingly ’perfect’ body for both women and men, it may facilitate the development of body image-related distress and eating disorders.

The choice to focus on adolescents and young adults between the ages of 14 and 29 responds to the objective of analyzing periods of life that, as evidenced by several studies, are particularly susceptible to discomforts both related to the perception of and satisfaction with body image and the problematic use of new technologies such as the Internet, social media, binge-watching, online gaming, etc. Adolescence represents, for every individual, a natural phase of research and construction of one’s physical and psychological identity and of comparison with peers and with ideal aesthetic models one would like to resemble, such as soccer players or other athletes, influencers, YouTubers, those in show business, and fashion personalities. In the complex process of recognizing and expressing one’s identity and in the construction and management of friendship and family relationships, which are undergoing profound transformation at this stage, adolescents who do not perceive a solid social context on which they can rely and/or who do not develop an adequate level of self-confidence to cope with stressful or difficult experiences are more likely to enact a series of dysfunctional behaviors that tend to manifest themselves mainly in the form of rigid control of their bodies as a tool for managing other aspects of their lives related to the definition of their identity, acquiring social recognition and support, and identifying their size in the world. If, during adolescence, the individual does not acquire the personal and social resources to complete this developmental process, these dysfunctional mechanisms may tend to persist and strengthen in adulthood. Research related to muscle dysmorphia is in its early stages and is characterized by several significant limitations, primarily the tendency to test mainly adult male subjects, particularly athletes, especially bodybuilders. Therefore, the choice to involve in our study a population of adolescents and young adults also of the female gender recruited outside predominantly sporting contexts, such as schools and universities, could allow for a broader observation of the phenomenon outside the restricted contexts generally examined by scientific research and offer new perspectives in the exploration of a phenomenon predominantly studied in male subjects, especially athletes and, among them, preferably bodybuilders and gym-goers.

## 2. Methods

### 2.1. Study Design

In the present study, we tested a mediation model, a type of analysis that aims to identify the mechanism underlying an observed relationship between one or more independent variables and one or more dependent variables, through the inclusion of a third or more explanatory variables, referred to as mediator variables, that can act directly on the dependent variable and influence the indirect effects of the predictor variables on it. Our model aims to test whether generalized self-efficacy (GSE) and problematic social media use (PSMU) are potential first- and second-level mediators, respectively, in the relationship between the independent variable represented by perceived social support (PSS) and muscle dysmorphia (MD), the dependent variable of the study.

### 2.2. Participants

The study targeted a population of both sexes aged 14–29 years, selected through a convenience sampling approach by involving Italian high schools and universities. In the demographic information section, participants were asked to indicate their male/female biological sex, gender identity with the possibility of expressing in addition to male/female a third non-binary choice, Italian or other origin, their educational qualification, caregivers’ educational qualification, and with whom they live. In light of these considerations, the only criterion for inclusion was age between 14 and 29 years, along with proficiency in the Italian language in order to understand the questionnaires well.

### 2.3. Setting

Participants were invited to take part in this study by completing self-report questionnaires administered online using Google Form software, version 0.8, which took about 15 min to complete and remained open for a period of two months. The form contained a description of the characteristics and purpose of the research, as well as the informed consent form that each participant had to sign to view the questionnaires. As for the group of adolescents, recruitment was performed through an e-mail invitation to some high school principals to participate in the research project. The e-mail described the characteristics and objectives of the study, recruitment methods, questionnaire administration methods, and rules regarding informed consent. The latter was delivered in paper form to pupils by teachers with a request to bring it back signed by parents or guardians. Students who did not deliver the signed informed consent form were automatically excluded from participating in the research. Since the questionnaires were to be administered online, it was required that each student have an electronic tool, smartphone, tablet, or PC. All questions were set as mandatory, so there are no missing data.

In accordance with the international guidelines of the Declaration of Helsinki 1964, last revision in 2000, and the code of ethics of the Italian Association of Psychology (AIP), participation in the questionnaire administration phase was subject to the signing of an informed consent form by each participant and parents/legal guardians in the case of minors. The study was approved by the Institutional Review Board (omitted for peer review). Participation was voluntary and no prizes or compensation were provided. Privacy was ensured at all stages of the research.

### 2.4. Variables

**Figure 1:**
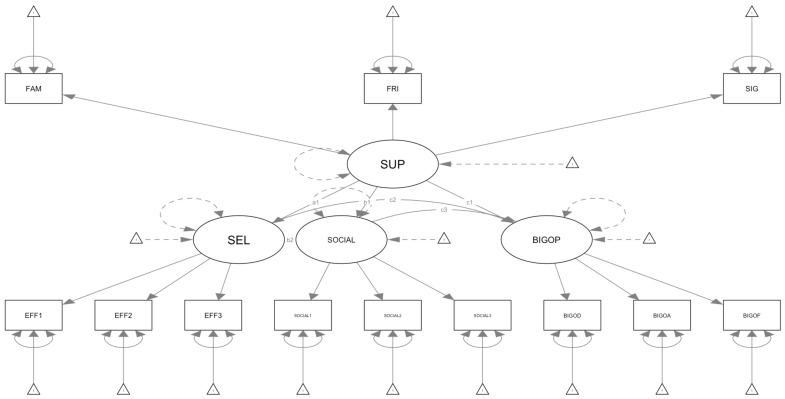
This figure delineates the Sem path of the selected model, determined through the parceling approach. Specifically, in the graph, circles represent latent variables, rectangles represent observed variables, and triangles represent measurement errors. SUP = perceived social support with FAM, FRI, and SIG representing its three constituent plots; SEL = general self-efficacy with EFF1, EFF2, and EFF3 representing its three constituent plots; SOCIAL = problematic use of social media with SOCIAL1, SOCIAL2, and SOCIAL3 representing its three constituent plots; BIGOP = muscle dysmorphia with BIGOD, BIGOA, and BIGOF representing its three constituent plots. Only the direct paths are shown in the figure to ensure a clear reading of the model. The paths a1 shows the direct effect between perceived social support and general self-efficacy, b1 shows the direct path between perceived social support and problematic use of social media, c1 the direct effect of perceived social support on muscle dysmorphia, b2 shows the direct path of general self-efficacy on problematic use of social media, c2 represents the direct effect of general self-efficacy on muscle dysmorphia and c3 the direct path of problematic use of social media on muscle dysmorphia.

All variables analyzed are quantitative, as evidenced by the use of exclusively self-report survey instruments. Mediation analyses were computed through Structural Equation Modeling (SEM). A structure with PSS, GSE, PSMU, and MD as latent variables was used to examine a model in which PSS is considered the predictor variable, GSE is the first-level mediator, PSMU is the second-level mediator, and MD is the outcome.The model can be observed in Figure 1.

### 2.5. Measurement

Perceived social support was measured with the Multidimensional Scale of Perceived Social Support (Downes et al., 2021; Zimet et al., 1988), which consists of 12 items divided into three subscales, each of which pertains to a different source of support, which has been shown to have strong factorial validity: (a) Family (i.e., “I get the emotional help and support I need from my family”), (b) Friends (i.e., “I have friends with whom I can share my joys and sorrows”) and (c) Significant Others (i.e., “I have a special person who is a real source of comfort to me”). Subjects are asked to indicate their degree of agreement with each of the items using a 7-point Likert scale (1 “completely agree”; 7 “completely disagree”).

General self-efficacy was measured through the General Self-Efficacy Scale (Sheehy et al., 2023), an instrument that evaluates the general sense of perceived self-efficacy, with the aim in mind to predict coping with daily hassles as well as adaptation after experiencing all kinds of stressful life events (i.e., “I am confident that I could deal efficiently with unexpected events”). The scale is designed for the general adult population, including adolescents. Responses are made on a 4-point scale. Each of the ten items that constitute the scale refers to successful coping and implies an internal-stable attribution of success. The responses to all 10 items are summed to yield the final composite score with a range from 10 to 40.

Problematic social media use was assessed through the Bergen Social Media Addiction Scale (Monacis et al., 2017), a six-item scale that assesses the past 12 months eventual misuse of Social Media (i.e., “Felt an urge to use SM more and more”). The scale was developed based on Griffiths (2005) with the objective of identifying the six core characteristics of addiction: salience, mood modification, tolerance, withdrawal, conflict, and relapse in relation to excessive and compulsive use of SM (e.g., Facebook, Instagram, etc.). The questionnaire uses a five-point Likert scale, ranging from 1 (very rarely) to 5 (very often). A cut-off of ≥19 was used to screen problematic social media use (PSMU).

Muscle dysmorphia was evaluated through the Muscle Dysmorphic Disorder Inventory (Santarnecchi & Dèttore, 2012), consisting of 13 items that the assess cognitive, behavioral, and emotional characteristics of muscle dysmorphic disorder. The MDDI items are divided into three factors: (1) drive for size (MDDI-DS, 5 items), which consists of questions regarding thoughts of being smaller, less muscular, and weaker than desired or a desire to increase size and strength (e.g., “I think my body is too small”); (2) appearance intolerance (MDDI-AI, 4 items), which consists of questions regarding negative beliefs about one’s body and the resulting appearance-related anxiety or avoidance of body exposure (e.g., “I wear baggy clothes so that people cannot see my body”); and (3) functional impairment (MDDI-FI, 4 items), which consists of questions designed to address the impairment associated with the symptoms of muscle dysmorphia (e.g., “I feel anxious when I miss one or more days of training”). The items are scored using a 5-point Likert-type response system ranging from 1 (never) to 5 (always). The total score can be derived from the sum of the subscales. Higher values reflect higher MD-related symptoms, and a cut-off point of 39 has been proposed and used in previous reports to identify clinically relevant MD.

Each variable was assessed using self-reports also validated in Italy and already administered and analyzed in samples of adults and adolescents demonstrating good levels of reliability and validity (Downes et al., 2021; Monacis et al., 2017; Santarnecchi & Dèttore, 2012; Schwarzer & Jerusalem, 1995; Sibilia et al., 1995; Zimet et al., 1988).These findings were corroborated by our model, as illustrated in Table 1.

### 2.6. Statistical Methods

IBM SPSS (Statistic Package Social Sciences) version 29 was used for preliminary statistical analyses including descriptive analysis of the sample’s main characteristics and correlational analysis of identified variables through estimation of Pearson’s product–moment correlation coefficients with a 95% bias-corrected and accelerated (BCa) confidence interval (CI). Internal reliability was verified by means of Cronbach’s α, which, according to (Nunnally, 1975) is considered satisfactory when values are greater than 0.70. The results are shown in Table 1.

R Studio version 4.3.2 with the lavaan package for R was employed to assess the factorial validity of the employed measures and the structural relationships between them.

The parceling approach was employed, whereby items from the questionnaires were randomly selected and aggregated. Parcels are more likely to satisfy the assumptions of normality and less susceptible to the influence of method effects (Little et al., 2002). The parceling approach was selected as it reduces model complexity, improves normality (parcels tend to have more normal distributions than individual items), increases long-term sustainability, and ensures more stable parameter estimates (Little et al., 2002). In the present study, three parcels were created for each observed and latent variable on a random basis. Regarding the evaluation of model fit indices, we referred to the guidance of Bentler and Bonett (1980) and Schermelleh-Engel et al. (2003), who recommend evaluating model fit using multiple indices: the Comparative Fit Index (CFI); the Tucker–Lewis Index (TLI); Standardized Root Mean Square Residuals (SRMRs); and the Root Mean Square Error of Approximation (RMSEA). Models with Comparative Fit Index (CFI) and Tucker–Lewis Index (TLI) values of 0.95 or greater and Standardized Root Mean Square Residual (SRMR) and Root Mean Square Error of Approximation (RMSEA) values of 0.08 or less are considered adequate (Bilgin & Taş, 2018; Schermelleh-Engel et al., 2003).

## 3. Results

### 3.1. Participants: Demographic Characteristics 

A total of 2325 study participants aged between 14 and 29 years (min 14, max 29 years, M ± SD 21.11 ± 4.94), of whom 1583 were female (68.1% of the total sample), completed the questionnaires. The age distribution of the sample population was as follows: 1571 young adults of both sexes aged over 18 years (67.6% of the total sample) and 754 adolescents of both sexes aged under 18 years (32.4% of the total sample). Regarding the sample size, we referred to the Monte Carlo power analysis for mediation models, which indicates that a minimum of 1000 participants is required to obtain a statistical power of 0.95 (Schoemann et al., 2017). Therefore, the sample size of this study is sufficient to ensure adequate statistical power.

### 3.2. Descriptive Data

With regard to other demographic characteristics, 96.8% of the participants indicated that their sociocultural background was Italian. With regard to gender, two distinct questions were posed: one concerning biological sex (male/female) and another pertaining to gender identity. In the latter category, 1.2% of respondents indicated that they identified as a gender other than male or female. The majority of participants (66.6%) reside with their parents or other equivalent figures, with a higher prevalence among those aged ≤17 years (79.5%) compared to those aged ≥18 years (60.3%). Additionally, 51.4% of participants’ mothers or equivalent figures and 47.7% of participants’ fathers or equivalent figures have attained a high school qualification.

### 3.3. Outcome Data

All data were analyzed using IBM SPSS (Statistic Package Social Sciences) version 29 and RStudio version 4.3.2 with the lavaan package.

All variables examined show significant correlations with each other (*p* < 0.01) and good internal reliability with Cronbach’s α greater than 0.70 (Nunnally, 1975). The results of the model are shown in Table 1.

### 3.4. Main Results

The results of Pearson’s correlation coefficient analysis suggest a high degree of significance in the correlation between all the variables examined, with particularly robust negative correlations observed between perceived social support and muscle dysmorphia (r = −0.310, *p* < 0.001) and the strongest positive correlation between problematic social media use and muscle dysmorphia (r = 0.373, *p* < 0.001), while no significant correlations were reported between the Drive for size and Functional impairment subscales of muscle dysmorphia with overall self-efficacy with values of (r = −0.007, *p* = 0.720) (r = −0.021, *p* = 0.323), respectively, correlations that were significant, however, in the Intolerance of appearance subscale (r = −0.244, *p* < 0.001) and in the total score of muscle dysmorphia (r = −0.140, *p* < 0.001). Finally, with regard to PSS, all of the variables analyzed show the strongest correlation with the Family subscale and the weakest correlation with the Friends subscale, and regarding MD, the most significant correlation appears to be with the Appearance intolerance subscale and the weakest correlation appears to be with the Drive for size subscale.

In constructing the mediation model, with regard to the independent variable consisting of perceived social support, we used the Multidimensional Scale of Perceived Social Support, which consists of three different scales: Family, Friends, and Significant Others, for each of which it is possible to measure a specific score or to assess a single score derived from the sum of the three different scores. In our model, we decided to use the variable in the form of a total score and to use the three scales as the three plots in our model. In fact, in this context, we were not interested in exploring the impact of each type of support on the mediator and dependent variables. The first-level mediator in our model is represented by general self-efficacy, for the assessment of which we used the General Self-efficacy scale which consists of a single scale and a total score; to construct three plots we divided the ten items into three groups. With regard to the second level mediator, which in our study consists of the problematic use of social media, measured by means of the Bergen Social Media Addiction Scale, an instrument consisting of a single scale and a total score, we divided the six items constituting it into three different groups in order to form the three plots to construct our model. Finally, with regard to the dependent variable represented by muscular dysmorphic disorder, we referred to the Muscular Dysmorphic Disorder Inventory, which consists of three scales with the possibility of calculating three different scores, one for each subscale, or a single score derived from the sum of the three specific scores; we opted for the latter option. The choice to use only the total score of the questionnaire stems from the intention to observe how the symptomatology of muscular dysmorphic disorder as a whole can be influenced by the predictor and mediator variables, whereas the evaluation of the three separate scores was not one of our analysis objectives. The construct thus identified proved to be sufficiently stable and robust, with satisfactory fit indices, as shown in Table 2.

The results of the model thus identified in the structural equation modeling analysis indicated that the direct effects of the predictor variable, i.e., PSS, on the symptomatology of muscle dysmorphia are significant and seem to confirm the results of previous studies and the initial hypotheses proposed in this research.

**Figure 2:**
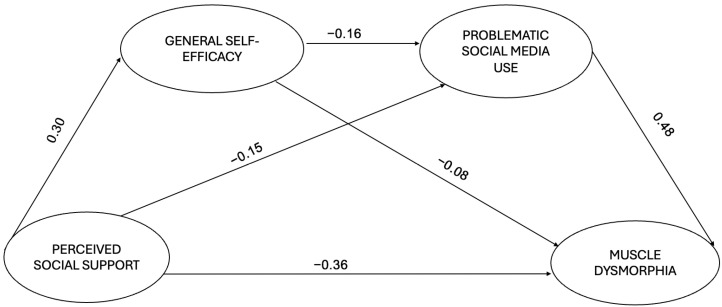
Structural model: Latent variables are circled. Arrows indicate the direction of the relationships between the analyzed variables. Note: Only direct paths are reported for clarity purposes; parcels were not reported for clarity purposes. For more details, see Table 3.

Furthermore, the indirect effects of PSS through GSE and PSMU on muscle dysmorphia symptomatology were both found to be statistically significant. Finally, the total effect, i.e., the sum of direct and indirect effects, of PSS on MD-related symptoms shows that PSS can influence GSE, which in turn can influence PSMU, and that both mediators influence muscle dysmorphia sequentially. The results thus seem to confirm our initial hypotheses and the hypotheses from the literature reviewed. The negative correlation between GSE and MD was also observed, just as the positive correlation between PSMU and MD directly and indirectly via GSE seems to be confirmed. More specifically, the hypothesized relevance of perceived social support, general self-efficacy, and problematic social media use on MD symptomatology seems to be supported. The results are presented in Figure 2 and Table 3.

### 3.5. Other Analyses

To examine the influence of gender on the proposed model, we incorporated the variable “gender” as a covariate in the regression analyses. The model was found to remain stable, with the relationships between the analyzed variables appearing unchanged, although the goodness-of-fit indices decreased slightly (CFI = 0.93; TFI = 0.90; SRMR = 0.08; RMSEA = 0.07). Therefore, although gender differences exist, they do not affect the pattern of relationships.

## 4. Discussion

Our study aimed to assess the role of perceived social support on the symptomatology of muscle dysmorphia through a mediation analysis in which perceived social support was tested as an independent variable to examine its direct and indirect effects, through a sequential mediation process, on the dependent variable represented by muscle dysmorphia. Our analysis is based on the recognition in the literature of the relevant role that perceived social support seems to have in improving individual well-being and in helping to cope with and overcome heterogeneous physical and psychological discomforts, particularly through the intervention of general self-efficacy, which is highlighted in the literature as an important factor in safeguarding individual psychophysical health, and which in fact represents the first-level mediator in our model. Furthermore, we hypothesized that problematic social media use would be negatively correlated with both individual perceptions of social support and generalized self-efficacy and positively correlated with symptoms of muscle dysmorphia. The results of our study, in line with the literature reviewed, seem to confirm the significance of the hypothesized relationships between the variables analyzed, thus demonstrating that the presence and intervention of certain psychological conditions and individual and social resources may influence the likelihood of developing MD-related symptoms. In particular, the perception of being able to count on solid social support from family, friends, and, more generally, the social context of reference, seems to be particularly important in this sense (Giordano et al., 2023). The problematic social interactions and relationships that adolescents and young adults engage in can lead to the development or persistence of dysfunctional behaviors (Cataldo et al., 2021; Saladino et al., 2024; Sandgren & Lavallee, 2023; Verrastro et al., 2024a; Verrastro et al., 2024b; Xie & Kim, 2022), with the risk that the need for social support, recognition, and appreciation from family, friends, and significant others in their lives will result in control over their bodies, diet and exercise. In this context, some studies suggest that an increase in social support on social media corresponds to a decrease in social support in real life, adding that while the latter seems to be associated with a reduction in depression, anxiety, and social isolation, social support on social media does not seem to protect against these psychological conditions (Monacis et al., 2017; Zhao et al., 2021). Understanding and assessing perceptions of social support in adolescents and young people at risk of muscle dysmorphia could provide health professionals with valuable information about their mental health, leading to more targeted interventions and support strategies. A similar argument can be made regarding the assessment of general self-efficacy, which, as shown in our study, could play an important role in the development and maintenance of dysfunctional use of new technologies, such as the Internet and social media. In particular, it has been shown that the use of these tools to search for content regarding exercise and weight loss strategies leads to greater dissatisfaction with body image and greater internalisation of the ideal of thinness (Favini et al., 2024; Hossain et al., 2024; Lippke, 2020; Schunk & DiBenedetto, 2021). This may be due to the use of this type of image as an ideal to strive for a slimmer and toned body in order to gain admiration and social approval, with potential negative effects on physical, psychological, and social well-being, such as reduced self-esteem and the onset of symptoms of depression and addiction (Imperatori et al., 2022; Li, 2019; Mancin et al., 2024). However, it is important to point out that our study assesses problematic social media use through the Bergen Social Media Addiction Scale, which, as previously reported, does not provide information on the specific content sought by users nor on the specific time of social media use but assesses the proposed criteria for behavioral addiction in terms of increasing time spent on social media, preoccupation, and inability to control social media use resulting in a negative impact on individual lives (Monacis et al., 2017; Ryding & Kuss, 2020; Sommantico et al., 2023). Regardless of the specific content, about which we have no information, the results of our study also show, consistent with the literature, that MD is positively associated with abuse of MS (Mancin et al., 2024; Ryding & Kuss, 2020), while it is negatively correlated with the perception of being efficient in coping with difficulties and unexpected events and having strong social support (Calaresi et al., 2024). The existing literature indicates that self-efficacy serves as a key protective factor in a multitude of psychological disorders (Favini et al., 2024; Liao et al., 2023; Sheehy et al., 2023). Therefore, enhancing self-efficacy should be an effective way to mitigate the negative effects of using social networking sites. Self-efficacy can be defined as the perceived ability to learn or perform actions at a certain level. Theory and research support the idea that self-efficacy is an important motivational construct capable of influencing choice, effort, persistence, and goal attainment, as revealed by, among others, Albert Bandura who, introducing his social cognitive and self-efficacy theories, proposed that self-efficacy and outcome expectations are fundamental to the initiation and maintenance of behavior (Bandura et al., 2003; Schunk & DiBenedetto, 2021; Tortosa Martínez et al., 2024).

Several studies have analyzed the interaction between social support and self-efficacy as a protective factor against psychological distress, linking it to students’ optimism, satisfaction with life, and better academic performance (Hossain et al., 2024; Tortosa Martínez et al., 2024), as well as a greater likelihood of overcoming traumatic events (Sheehy et al., 2023) and coping with serious physical illness (Dinh & Bonner, 2023; Iovino et al., 2023; Iwanowicz-Palus et al., 2021). Thus, it can be argued that self-efficacy and perceived social support represent a knowledge of oneself as capable and of the world as friendly, which in turn can lead to an assessment of the future as potentially beautiful and fruitful and improved social and psychological functioning and health status (Czyżowska & Gurba, 2021; Meshi & Ellithorpe, 2021). Particularly for younger people, internalizing this self-confidence may reduce the importance of external assessments, such as societal pressures or criticism related to physical appearance. Indeed, as noted above, the literature emphasizes expectations of personal efficacy as determining the initiation of coping behaviors, as well as the level and duration of effort in the face of obstacles and negative experiences. Self-efficacy beliefs are dynamic and result from the interaction between the individual and the environment. They can be reinforced through direct experience, as consistent engagement in perceived challenging activities can lead to mastery experiences, which in turn increase self-efficacy and reduce defensive behaviors, or indirectly through modeling, which involves observing the successful experiences of similar individuals, such as peers and classmates (Giordano et al., 2023). In conclusion, the results of the present study are consistent with previous research that perceived social support appears to be a significant factor correlated with better mental health, both in terms of improved psychological resources and social skills, i.e., self-efficacy, and as a protective factor in the development and maintenance of psychological distress, such as maladaptive use of social media and distorted perceptions of body image, particularly physical fitness and musculature.

## 5. Conclusions

The results of our investigation confirm the initial hypotheses and assumptions of the literature on the subject, emphasizing the central role that perceived social support and general self-efficacy can play in mental well-being, both in dysfunctional behavior associated with the misuse of new technologies—in this case, the problematic use of social media—and in the discomforts related to an erroneous perception of one’s own body image, represented here by muscular dysmorphia. However, it should be noted that the cross-sectional nature of the study and the exclusive use of self-reported instruments entail some limitations to the results. Firstly, the cross-sectional design inevitably precludes the possibility of drawing causal inferences between the variables considered. In fact, due to their inherent nature, cross-sectional designs can only represent a snapshot of a moment in time of the trajectory and a much broader functioning of the variables under examination. In order to confirm the validity of the selected mediation model, it is advisable for future research to involve longitudinal or experimental designs, which would provide a more complete and detailed understanding of the interaction between the variables under examination. Indeed, such models allow for a better evaluation of the results derived from the cross-sectional model, allowing causal inferences to be drawn and providing stronger evidence of the existence and functioning of the hypothesized relationships between the analyzed variables. Another major limitation is the exclusive use of self-report instruments without qualitative diagnostic or confirmatory interviews. Although self-report instruments are undoubtedly the simplest and fastest method of data collection, they are not without their inherent limitations. Self-reported responses may be exaggerated, respondents may be too embarrassed to reveal personal details, and various biases may influence the results. These include social desirability and social esteem biases, indulgence biases, acquiescence biases, and the need for consistency and rationality. In addition, the inability to understand certain words and the presence of negative, structured, or ambiguously or ambivalently phrased sentences may lead to difficulties in answering for those participants who read the questions quickly or distractedly or who may be influenced by the framing effect. Finally, the interference of memories and emotions in the interpretation of events, especially those from the past, may also be a source of common and sometimes significant biases in participants’ approach to questionnaires. These instruments should be improved by incorporating qualitative methods and/or clinical interviews to reduce their typical limitations. Again, a useful strategy to address these constraints is the implementation of a longitudinal design to mitigate the inherent biases associated with self-report interviews and to gain a better understanding of the nature and directions of the relationships between the variables under study. In addition, it would be advantageous to expand the study population through the involvement of a sample of individuals older than 17 years, particularly emerging and young adults, in order to obtain a more complete understanding of the phenomenon under analysis and to monitor the characteristics and dynamics it may acquire in the transition between adolescence and adulthood. Observation of a population of other age groups would allow us to observe the evolution of the phenomenon or its possible later relapse episodes in relation to maladaptive personality traits, which are more stable and defined in adulthood, and to delve into the diachronic dimension of the role of PTSD and/or DSO symptomatology related to traumatic and adverse events experienced later in life or, if they occurred in childhood and adolescence, to verify their effects at a later date.

In addition, it might be interesting to include a sample of adults older than 29 years in the research to allow a broader view of the phenomenon analyzed and how the dynamics related to it work after adolescence and young adulthood, to find out if there are specific differences or characteristics in the development, evolution, and maintenance of this distress in relation to age and different stages of life. Moreover, although the questionnaire utilized to identify the symptomatology of muscle dysmorphia (i.e., the Muscle Dysmorphia Disorder Inventory) has been successfully validated, including in Italy, in both male and female populations, with the objective of gaining a more profound and comprehensive understanding of the prevalence, impact, and characteristics of the symptomatology of muscle dysmorphia in a female population, it is recommended that clearer and more unambiguous definitions be employed in certain items of the MDDI. In this regard, it would be beneficial to provide more detailed specifications of terms that could be interpreted in a biased or unclear manner. For instance, the term “big” could be replaced or augmented with the more precise term “muscular”, which could help to prevent any potential misunderstanding. This could prove crucial for future research endeavors aimed at a more profound and comprehensive assessment of gender-based disparities in the manifestations and characteristics of muscle dysmorphic disorder. It may also be of interest to include information on participants’ physical activity in future studies, with a view to identifying any differences or points of contact between those who engage in physical activity or sport and those who do not.

Despite the limitations mentioned above, the results of our study may suggest to researchers and clinicians dealing with discomfort related to body dysmorphia, also in the form of muscle dysmorphia, and discomfort related to the misuse of new technologies, such as the Internet, smartphones and especially social media, that interventions aimed at improving social and relationship skills may be effective in preventing MD-related discomfort, especially in adolescence and young adulthood, when early recognition of the symptoms of psychological distress could help prevent the adoption of dysfunctional behavior, thus offering adequate support at a time when aspects of growth such as the formation of individual identity and the management of social relationships are often perceived as out of control. From this perspective, health professionals working with individuals at risk for or diagnosed with MD might consider interventions aimed at strengthening social support networks and promoting healthier patterns of social media use. Interventions could include psychoeducation about the potential negative effects of excessive social media use, as well as skills training in developing self-efficacy and building and maintaining supportive offline relationships.

## Figures and Tables

**Table 1:** Descriptive analyses, reliability, and correlations.

	M	SD	*S*	*K*	*α*	1	2	3
1. Perceived Social Support	5.59	1.09	−1.00	0.79	0.91	-	-	-
2. General Self-Efficacy	4.72	1.12	−0.23	−0.04	0.94	0.28 *	-	-
3. Problematic Social Media Use	2.39	0.93	0.44	−0.46	0.84	−0.16 *	−0.18 *	-
4. Muscle Dysmorphia	2.07	0.70	0.66	0.22	0.82	−0.31 *	−0.14 *	0.37 *

Note: *N* = 2325. * *p* < 0.01.

**Table 2:** Goodness-of-fit indices of the measurement models.

	χ^2^	*p*	CFI	TFI	SRMR	RMSEA
Fit Indices	(48) = 576.39	<0.01	0.96	0.95	0.06	0.07

**Table 3:** Path estimates of the structural model, SE, and 95% CIs. The following table shows the values of standardized beta coefficients, standardized errors, *p*-values, and maximum and minimum levels of confidence intervals.

Direct Effect	*β*	*p*	SE	CI LL	CI UL
PSS → GSE	0.30	<0.001	0.04	0.40	0.56
PSS → PSMU	−0.15	<0.001	0.03	−0.20	−0.09
GSE → PSMU	−0.16	<0.001	0.02	−0.13	−0.07
PSS → MD	−0.36	<0.001	0.02	-0.20	−0.13
GSE → MD	0.08	0.01	0.01	0.01	0.04
PSMU → MD	0.48	<0.001	0.02	0.19	0.27
Indirect effect via GSE					
PSS → MD	0.02	0.01	0.004	0.003	0.02
PSS → PSMU	−0.05	<0.001	0.01	−0.06	−0.03
Indirect effect via PSMU					
GSE → MD	−0.08	<0.001	0.004	−0.03	−0.02
PSS → MD	−0.07	<0.001	0.01	−0.05	−0.02

Note: GSE = general self-efficacy; PSS = perceived social support; PSMU = PSMU; MD = MD; β = standardized beta coefficient; *p* = level of significance; SE = standard error; BC 95% CI = confidence interval; LL = lower limit; UL = upper limit.

## Data Availability

The raw data supporting the conclusions of this article will be made available by the authors upon request.
